# Transfabric closure of a peridevice leak using a PFO occluder after transcatheter left atrial appendage closure: A case report

**DOI:** 10.1016/j.hrcr.2025.08.025

**Published:** 2025-08-26

**Authors:** Karthik Venkatesh Prasad, Alejandro José Quiroz Alfaro, Austin Urvina, Elsheikh M. Abdelrahim, James E. Stone

**Affiliations:** 1 Department of Electrophysiology, North Mississippi Medical Center, Tupelo, Mississippi; 2 Department of Internal Medicine, North Mississippi Medical Center, Tupelo, Mississippi

**Keywords:** Peridevice leak, Endovascular treatment, Left atrial appendage closure, Atrial fibrillation, Amplatzer Talisman, Patent foramen ovale occluder, WATCHMAN device, Experimental treatment, Cardiac electrophysiology, Left atrial appendage

**Key Teaching Points**

• The transfabric closure of a peridevice leak (PDL) using an Amplatzer Talisman patent foramen ovale occluder seems to be a safe and feasible alternative to other endovascular treatment options, including coils and vascular plugs.
• PDLs remain a challenging ongoing issue because there is no clear consensus on their significance, definition, or management approaches.
• Endovascular treatment of PDLs should be carefully considered on a case-by-case basis, especially because PDLs are associated with thromboembolic complications, regardless of the PDL size.

## Introduction

The transcatheter left atrial appendage closure (LAAC) remains a safe alternative to anticoagulation for reducing the risk of thromboembolic events in patients with nonvalvular atrial fibrillation (NVAF) who are intolerant to oral anticoagulants (OACs).^1^^,^^2^ The PROTECT AF trial demonstrated that LAAC with the WATCHMAN device (Boston Scientific) met the criteria for both noninferiority and superiority in preventing the combined outcomes of cardiovascular death, stroke, and systemic embolism compared with warfarin.^3^ In addition, the WATCHMAN device was superior in reducing cardiovascular and all-cause mortality compared with warfarin.^3^

More recently, a multicenter, retrospective matched cohort study by Khalid et al^4^ compared the rates of major bleeding and ischemic strokes in patients with NVAF who either underwent LAAC using a WATCHMAN device or received direct OAC therapy after a major bleeding event. The authors reported that the WATCHMAN cohort had significantly lower rates of major bleeding events, transient ischemic attacks, and ischemic strokes.^4^ At the same time, there was no significant difference in hemorrhagic strokes between the 2 groups.^4^

Although transcatheter LAAC has advantages over OAC, it can lead to both intra- and postprocedural complications. The most common intraprocedural complications include pericardial effusion and tamponade, whereas device-related thrombus and peridevice leak (PDL) are among the most frequent postprocedural complications.^1^^,^^5^ Depending on the study, PDL has a variable incidence at 45 days after LAAC, ranging from 26.6% to 54%; this complication is significant because it has been associated with an increased risk of ischemic stroke and transient ischemic attack.^6^

Here, we present a case of a significant PDL after transcatheter LAAC that required endovascular closure owing to the patient’s intolerance to OAC or dual antiplatelet therapy (DAPT) caused by recurrent episodes of gastrointestinal bleed (GIB).

## Case presentation

Our patient is a 63-year-old gentleman with a medical history of paroxysmal NVAF and premature coronary artery disease requiring bypass surgery with intraoperative mitral valve ring annuloplasty for severe functional/secondary mitral regurgitation. With persistent severe ischemic cardiomyopathy with an ejection fraction of <35% on goal-directed medical therapy, he had a single-chamber defibrillator implanted for primary prevention of sudden cardiac death. He also has chronic kidney disease stage 3A.

OAC was being used for primary stroke prevention from NVAF using rivaroxaban, based on a CHA_2_DS_2_-VASc score of 3. However, this was complicated by recurrent episodes of GIB from small intestinal arteriovenous malformations, resulting in symptomatic anemia requiring blood transfusions on more than 1 occasion. After discussing the pros and cons and based on a HAS-BLED score of 3, the patient underwent transcatheter LAAC with a 40 mm WATCHMAN FLX Pro device (Boston Scientific).

**Figure 1:**
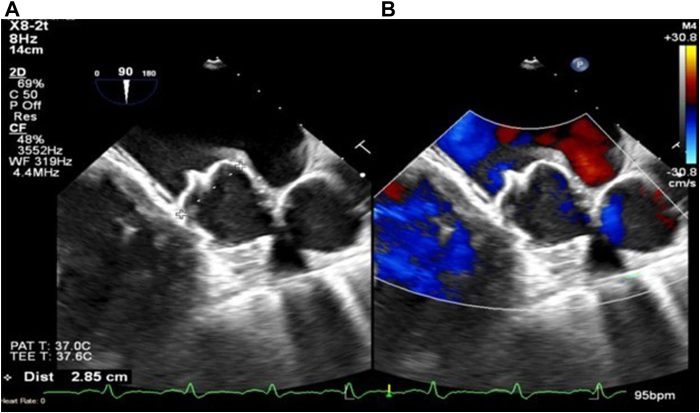
Intraprocedural TEE during the WATCHMAN FLX Pro device implant. 90° TEE showing 2D (**A**) and color Doppler images (**B**) demonstrating no peridevice leak after the release of the 40 mm WATCHMAN FLX Pro device. 2D = 2-dimensional; TEE = transesophageal echocardiogram.

The implant procedure was rather challenging owing to difficult coaxial access after transseptal puncture and a large left atrial appendage (LAA) size (maximal diameter of 27.8 mm; maximal length of 25.7 mm). The puncture was performed in the middle of the atrial septum using the bicaval long axis and the short axis view (“mid-mid”) of the transesophageal echocardiogram (TEE) as guidance. The closure device was deployed in a stable ostial location with appropriate compression (25%) and stability with no PDL (Figure 1 and Supplemental Video 1). The procedure was performed by a single operator under deep sedation (propofol administered by an anesthesiologist) and TEE guidance, per our institution’s protocol.^1^ Intraprocedural left atrial (LA) pressure was 22 mm Hg. After the procedure, he was discharged on DAPT using 81 mg aspirin and 75 mg clopidogrel every day.

Six weeks later, he was hospitalized again for GIB with hemorrhagic shock requiring the use of vasopressors and blood transfusions. Once stabilized, he underwent a routine 45-day follow-up TEE to evaluate the LAAC device. TEE revealed a 3.6 mm PDL adjacent to the LAA ridge, with flow also noted on the color Doppler study. This was visualized in multiple views (Supplemental Video 1). Despite evidencing the residual PDL, the device compression remained adequate at 25%. Given that this was the largest transcatheter LAAC device available and the patient still had residual significant PDL with continued inability to tolerate DAPT or OAC (owing to recurrent GIB), the option of endovascular closure of his PDL was presented to further mitigate his stroke risk. The investigational nature of the procedure was explained to the patient (including risks and benefits), to which the patient agreed.

The procedure was performed under general anesthesia. An intravenous heparin bolus was given upfront (100 units/kg) and periodically to maintain an activated clotting time of >250 seconds throughout the procedure. Using a right femoral venous approach, transseptal left heart catheterization was performed via a Lamp transseptal sheath and BRK needle (Abbott), although the initial “mid-mid” puncture was guided by TEE and fluoroscopic images. A J wire was then advanced into the left superior pulmonary vein, and the sheath was later advanced over it. With the dilator and sheath positioned in the left superior pulmonary vein, the wire was removed, and the same needle was advanced carefully ensuring it stayed inside the sheath.

**Figure 2:**
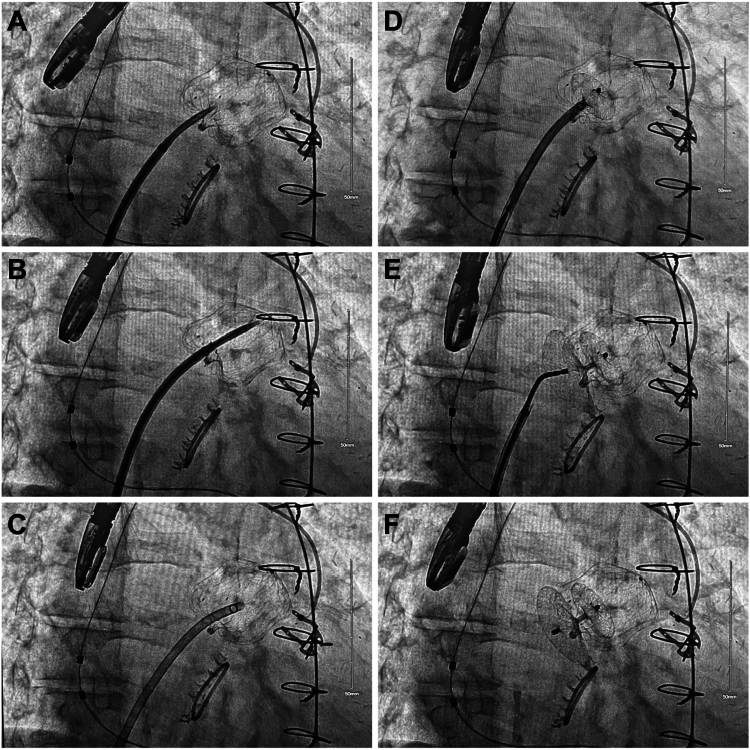
Right anterior oblique fluoroscopy projections during the peridevice leak closure via transfabric puncture approach. Fluoroscopy projections showing the over-the-needle advancement of a dilator into the WATCHMAN FLX Pro device (**A, B, C**). Fluoroscopy projections showing the uneventful release of the Amplatzer Talisman patent foramen ovale occluder via the lamp sheath (**D, E, F**).

Under 2- and 3-dimensional TEE and fluoroscopy guidance, the fabric of the WATCHMAN FLX Pro device was punctured (favoring the side of the PDL by the LAA ridge), and carefully, a dilator and the sheath were advanced over the needle into the WATCHMAN FLX Pro. Once the Lamp sheath was inside the WATCHMAN FLX Pro, the dilator and the needle assembly were removed (Figure 2).

A 35 mm Amplatzer Talisman patent foramen ovale (PFO) occluder (Abbott) was prepped and inserted via the Lamp sheath. The LA disc (smaller disc) was deployed inside the WATCHMAN FLX Pro. Upon evaluating the position under fluoroscopy and TEE, this disc was pulled back onto the inner side of the WATCHMAN FLX Pro device (Figure 2D), after which a tug test was done. Then, the Lamp sheath was peeled back to expose the right atrial disc (the larger disc) of the device, which landed perfectly on top of the LAA ridge and covered the WATCHMAN FLX Pro device completely. Another tug test was performed after the right atrial disc was completely exposed, just before releasing the device. TEE images, including 2-dimensional, 3-dimensional, and color-flow Doppler, demonstrated excellent positioning. The previously noted 3.6 mm PDL was no longer present (Supplemental Video 1). The Amplatzer Talisman PFO occluder was released uneventfully. The sheath was removed and hemostasis at the access site was achieved with a figure-of-8 suture. The patient tolerated the procedure well and was discharged home the subsequent day on single antiplatelet therapy with 81 mg aspirin only. Six weeks after the PFO closure device implant procedure, a follow-up TEE showed no persistence of the PDL.

## Discussion

Despite the association of PDL with thromboembolic complications, currently, there is no consensus regarding the definition of PDL, the optimal imaging method for its detection, or the grading of its severity.^7^ According to a meta-analysis by Mostafa et al,^8^ most studies define PDL as a color jet around the occlusion device observed in at least 2 views during the postprocedural TEE.

Nevertheless, it has been observed that, in some patients, the LAA does not fully become thrombosed after LAAC, which can result in a residual connection between the LA and the LAA.^7^ This residual connection is attributed to a leak in the fabric of the closure device, even in the absence of visible communication between the LA and its appendage.^7^ In our case, there was no leak involving the fabric of the closure device during the 45-day follow-up TEE but rather a true PDL owing to the inadequate size of the LAAC device compared with the large LAA size.

Initial clinical trials considered a PDL of ≤5 mm as “sufficient” LAAC; however, analysis of the outcomes from the data from such trials suggests that even PDLs of ≤5 mm have been associated with an increased risk of ischemic stroke and systemic embolism.^9^ The cutoff for a “large” or “significant” PDL is currently lacking consensus because different studies assign different cutoffs varying from >3 mm to >5 mm.^1^^,^^8^

Bhuta et al^10^ reported that up to 70% of PDLs regress over time, regardless of size, based on a single-center study of 139 patients with PDLs (mean leak size 3.2 ± 1.4 mm) after LAAC with the WATCHMAN FLX (Boston Scientific). While awaiting regression of these PDLs, patients were on OAC for variable periods of time. This would have been an unacceptable option for our patient, given his history of recurrent GIBs.

A few case series have demonstrated the feasibility and safety of the endovascular closure of PDLs of ≥3 mm using vascular plugs, coils, or another LAAC device.^11^, ^12^, ^13^ In our experience, transfabric closure of a PDL using a PFO occluder has been rarely reported. In this case, we considered it a suitable alternative to other endovascular treatments given that up to 18.9% of patients undergoing endovascular treatment with vascular plugs and coils had persistent PDLs, as reported in an international, multicentric study.^14^ The reinitiation of oral anticoagulation was also absolutely contraindicated in our patient.

## Conclusion

Transcatheter LAAC devices and techniques have evolved considerably; however, PDL (and its associated risk of stroke) is a limitation that warrants reinitiation of OAC. Preprocedural imaging, proper implant technique, intraprocedural imaging, and optimal choice of LAAC device can help limit postprocedural PDL; however, certain anatomic factors can be unavoidable. Endovascular treatment of PDL should be considered on a case-by-case basis, particularly in patients for whom reinitiation of OAC is contraindicated. When feasible, the transfabric puncture approach to closure of a PDL using a PFO occluder can be 1 such endovascular treatment approach to treat PDL. Although our case had an acutely successful outcome, this was a single case report of an experimental approach. Further follow-up data on such approaches are needed to study their long-term efficacy and safety.

## Disclosures

Although the authors did not receive specific funding or sponsorship for this manuscript’s publication, Dr Prasad was compensated for performing the peridevice leak endovascular closure as an electrophysiologist. The remaining authors declared no conflict of interest.

## Appendix Supplementary Data

Supplementary Video Legend

Supplementary Video Video showing the intraoperative LAAC TEE findings (**A**); 45-day follow-up findings on TEE (**B**) and, post-PDL closure findings on TEE (**C**), respectively
