# Supplementary material for: Transfabric closure of a peridevice leak using a PFO occluder after transcatheter left atrial appendage closure: A case report
Source: HeartRhythm Case Rep. 2025 Aug 26;11(11):1202–5. doi: 10.1016/j.hrcr.2025.08.025 (PMC12666904; doi:10.1016/j.hrcr.2025.08.025)
Supplement: Supplementary Video Legend [file mmc1.docx]

**Video 1:** Video showing the intraoperative LAAC TEE findings (**A**); 45-day follow-up findings on TEE (**B**) and, post-PDL closure findings on TEE (**C**), respectively.
